# A Real-World Effectiveness of Subcutaneous Immunotherapy on the Cost of Medication, Allergic Rhinitis, and Asthma Exacerbations, as well as Upper Respiratory Tract Infection in Subjects with Allergic Rhinitis with or without Asthma: A Retrospective Pilot Study

**DOI:** 10.3390/medicina57111229

**Published:** 2021-11-11

**Authors:** Chaicharn Pothirat, Warawut Chaiwong

**Affiliations:** Division of Pulmonary, Critical Care and Allergy, Department of Internal Medicine, Faculty of Medicine, Chiang Mai University, 110 Inthavaroros Rd. Sriphum, Maung Chiang Mai, Chiang Mai 50200, Thailand; warawut.chai@cmu.ac.th

**Keywords:** allergy, asthma, allergen, immunotherapy, cost

## Abstract

*Background and Objectives*: Real-world studies are limited regarding the effectiveness of SCIT on allergic rhinitis (AR) with and without asthma and the cost of medication in Thailand. Moreover, limited data exist regarding the effectiveness of SCIT on worldwide upper respiratory tract infection (URTI). Therefore, the objective of this study was to compare the medication costs, rate of AR and asthma exacerbations, and rate of URTI in AR with or without asthma subjects before and during three years after receiving the maintenance phase of SCIT, compared with a standard usual care (SUC) group. *Materials and Methods*: A real-world retrospective study was conducted in AR subjects with or without asthma. From January 2001 to December 2018, 24 subjects with or without asthma received SCIT added to SUC, and 16 subjects were treated with SUC only at the Allergy and Chest Clinic of Chiang Mai Ram Hospital, Chiang Mai, Thailand. The cost of medication was recorded. AR and asthma exacerbations and URTI events were also collected. *Results:* From between-group comparisons, the cost of medication (THB) in the SCIT group at the one-, two-, and three-year follow up was significantly lower (587.4 (348.3–1374.6) vs. 1562.4 (1315.1–1857.3), *p* < 0.001, 501.2 (302.9–839.0) vs. 1728.3 (1190.0–2236.1), *p* < 0.001, and 372.4 (284.8–752.4) vs. 1500.3 (1217.9–1748.9), *p* < 0.001, respectively)), and AR and asthma exacerbations were significantly reduced at the three-year follow-up. From within-group comparisons, the cost of medication (THB) and AR and asthma exacerbations were significantly lower in the SCIT group at the one-, two-, and three-year follow-up. The URTI event was significantly reduced in the SCIT group at the two- and three-year follow-up. *Conclusions*: SCIT in subjects with AR with or without asthma was associated with a significantly reduced cost of medication, rates of AR and asthma exacerbations, and URTI events in the long term.

## 1. Introduction

Allergic rhinitis (AR) is a common disease, characterized by paroxysms of sneezing, rhinorrhea, and nasal obstruction, often accompanied by itching of the eyes, nose, and palate [1]. In Chiang Mai, Thailand, the prevalence of AR was reported by Pothirat et al. [2]. They reported the prevalence of AR at 41.6% [2]. They also reported that the most common aeroallergen for AR was mites mixed followed by cockroach mixed and grass pollen [3].

Subcutaneous immunotherapy (SCIT) is a method capable of altering the natural courses of IgE-mediated allergic diseases [4]. It is effective in preventing sensitization to new allergens, reducing the risk of developing asthma, and maintaining its therapeutic effects upon treatment completion [4,5,6]. In previous studies, SCIT was proven to significantly decrease symptoms [7,8,9,10,11,12], exacerbations [13], the medication used [9,10,11,14], and medication costs [15,16,17] in subjects with AR and asthma. To the best of our knowledge, real-world studies regarding the effectiveness of SCIT on the cost of medication, AR and asthma exacerbations, and upper respiratory tract infection (URTI) events in Thailand are still limited [18]. Therefore, the primary objective of this study was to compare the cost of medication, rates of AR, and asthma exacerbation before and during a three-year follow-up after receiving the maintenance phase of SCIT added to standard usual care (SUC) for AR and AR with asthma subjects to those received SUC only. The SUC measures consisted of pharmacotherapy and environmental control of specific allergen, climate (temperature and humidity), and air pollutants.

A previous study indicated that adults with AR experience significantly more respiratory tract infections than non-AR adults [19]. From the clinical point of view, it is important to recognize that AR subjects are a risk group for respiratory infections. To the best of our knowledge, limited data exist regarding the effectiveness of SCIT on URTI. Therefore, the secondary objective of this study was to evaluate the rate of URTI before and three years after receiving either SCIT or SUC in subjects with AR and AR with asthma.

## 2. Materials and Methods

### 2.1. Study Design

A real-world retrospective case-controlled study was conducted on AR with or without asthma subjects who were still symptomatic despite optimum pharmacotherapy and environmental control. All subjects had regular visits during SCIT protocol (Table 1) and had no financial problems with the cost of medication. From January 2001 to December 2018, eighty-seven subjects were treated with SCIT. However, 40 subjects were followed up for at least three years. AR with or without asthma (*n* = 24) and AR with asthma (*n* = 15) subjects received SCIT for either mite mixed, cockroach mixed, or grass-pollen allergens added on SUC (SCIT group) and AR with or without asthma (*n* = 16) and AR with asthma (*n* = 10) subjects were treated with SUC only (SUC group) at the Allergy and Chest Clinic of Chiang Mai Ram Hospital, Chiang Mai, Thailand. The AR and asthma medication treatment in SUC care was prescribed according to up-to-date Allergic Rhinitis and its Impact on Asthma (ARIA) and the Global Initiative for Asthma (GINA) guidelines at each period. The standard commercial therapeutic agents (Alk-Abello, Lincoln Diagnostics, Dallas, Tx, USA), including (mite mixed (Dermatophagoides pteronyssinus (DP) Dermatophagoides farina (DF) (10,000 AU/mL), cockroach mixed (American, German) (1:20 *w*/*v*), Bermuda grass (10,000 PNU/10 mL), Johnson grass (1:10 *w*/*v*)). The subjects in the SCIT group had gone through an induction phase lasting approximately 17 weeks followed by a maintenance phase lasting three years after the induction phase. During the induction phase and maintenance phase, the subjects were vaccinated once a week (±2 days) and every 4 ± 1 weeks, respectively. Therapeutic extract doses were gradually increased up to the maximum tolerated dose according to our SCIT protocol (Table 1). After each injection, all subjects were instructed and observed for systemic reactions for at least 30 min. If the systemic reactions occurred after 30 min, the subjects could report directly to the physician. This study was approved by the Ethics Committee of the Faculty of Medicine, Chiang Mai University (study code: MED-2562-06485, date of approval: 8 August 2019) in compliance with the Declaration of Helsinki.

### 2.2. Data Collection

The cost of medication used was calculated applying pharmacy recommended average retail prices of the government-based Hospital Formulary 2008. The cost of medication in this study includes anti-allergy drugs, asthma drugs, systemic and oral steroids, antibiotics, and the cost of SCIT. The registry only included person-specific data for the latest three years. AR and asthma exacerbations requiring systemic corticosteroids and URTI events requiring antibiotics were also collected before and during three years of follow-up for the SCIT and SUC groups based on each patient’s medical records. Adverse systemic reactions from SCIT were also reviewed. This study utilized de-identified retrospective medical data. Therefore, this study did not require informed consent. Moreover, the released data were all anonymized and the researchers could not identify the participants.

### 2.3. Statistical Analysis

Data are presented as mean ± standard deviation (SD) unless stated otherwise. The category data are reported in number and proportion. Baseline differences and clinical characteristics between SCIT and SUC groups were analyzed using an independent T-test and Fisher exact test for continuous data and category data, respectively. Mann–Whitney U test was used for analyzing the differences in nonparametric data between SCIT and SUC groups. The Wilcoxon signed-rank test was used for comparing the nonparametric data at the first year, second year, and third year versus baseline in each group. Statistical analysis was performed using a STATA version 16 software package (StataCorp, College Station, TX, USA).

## 3. Results

Baseline clinical characteristics of the subjected in SCIT and SUC groups were comparable except that the SCIT group had a longer duration of AR or asthma disease than the SUC group (11.5 years (9.0–18.3) vs. 7.5 years (5.0–8.0), *p* < 0.001). More data are shown in Table 2. The systemic reactions (e.g., generalized pruritus, urticaria, flushing, angioedema, shortness of breath, wheezing, sneezing, rhinorrhea, nasal pruritus, nasal congestion, itchy throat, cough conjunctivitis, nausea, and headache) from SCIT were found in only 11 events out of 2092 injections (0.0053 per injection) and no anaphylaxis occurred.

The medication cost between SCIT and SUC groups throughout the study periods in the total subjects and AR with asthma subjects is shown in Table 3. The costs of medication at baseline were comparable between the SCIT and SUC groups. During the maintenance phase for three years, the medication cost was significantly lower in SCIT compared with the SUC groups. The medication cost was also significantly reduced compared with the baseline in the SCIT group. In the SUC group, there was no significant change in the medication cost throughout the three-year follow-up periods. More data are shown in Table 3.

The rates of AR and asthma exacerbation requiring systemic corticosteroids between the SCIT and SUC groups throughout the three-year follow-up periods in the total AR and AR with asthma subjects are shown in Table 4. At the baseline, the AR and asthma exacerbations were higher in the SCIT group compared with the SUC group, but not significantly different. During the maintenance phase of three years, the AR and asthma exacerbations were lower in the SCIT group compared with the SUC group, but not significantly different. The AR and asthma exacerbations were significantly reduced compared with the baseline in the SCIT group. In the SUC group, there was no significant change in exacerbations of AR or asthma throughout the three years of the study periods. More data are shown in Table 4.

Concerning the URTI events requiring antibiotics between the SCIT and SUC groups throughout the study periods in total subjects and AR with asthma, subjects are shown in Table 5. At baseline, the URTI events were higher in the SCIT group compared with the SUC group, but not significantly different. The URTI events were significantly reduced compared with the baseline in the SCIT group (at the two- and three-year follow-up periods for total subjects and the three-year follow-up period for AR with asthma subjects). In the SUC group, there was tendency for URTI to increase throughout the three years of the study periods, but it did not reach statistically significant differences. More data are shown in Table 5.

## 4. Discussion

Our real-world study showed the effectiveness of SCIT in subjects with AR and AR with asthma. During the three-year maintenance phase of SCIT, the medication cost in the SCIT group was significantly reduced, and the rates of AR and asthma exacerbations and the rate of URTI also decreased.

Our results showed that the medication cost was significantly lower in SCIT compared with the SUC groups since the first year after receiving SCIT. Moreover, the cost of medication per month was significantly reduced compared with the baseline in the SCIT group throughout three years of follow-up (1822.5 THB to 372.4–587.4 THB and 1938.5 THB to 563.9–644.6 THB in total subjects and AR with asthma subjects, respectively). Our results were supported by previous findings indicating that SCIT in AR with or without asthma has cost-saving effects owing to a reduction in the use of the anti-allergic medication and asthma medications [16,17,20].

Our results also showed that the AR and asthma exacerbations that required systemic corticosteroids were significantly reduced compared with the baseline in the SCIT group. The rate of AR and asthma exacerbations was reduced from baseline to the one-, two-, and three-year follow-up periods (the median was reduced from 1 to 0 times per year and 1.5 to 0 times per year in total subjects and AR with asthma subjects, respectively). Our results support previous findings that the number of AR or asthma exacerbations significantly decreased among SCIT patients compared with controls [13,21]. For example, Pifferi found that the number of asthma exacerbations significantly decreased in SCIT patients compared with controls, in which the difference was observed after the first year and remained significant at the end of the three years [21]. They also found that the number of asthma exacerbations significantly decreased in SCIT patients from the baseline to one to three years of follow-up (8 vs. 1–2 times per year) [21].

A previous study demonstrated that adults with atopic disease had a significantly increased risk of URTI, including the common cold, sinusitis, tonsillitis, and otitis media, with an adjusted risk ratio (RR) of 1.55 (95% CI; 1.14, 2.10) [19]. The higher rate of URTI may be caused by the T-helper type 1 (Th1) response that plays an important role in protection against infectious agents, mainly through interferon-gamma [22]. Th1 has been claimed to diminish in allergic individuals, leading to impairment of host defense responses against microbial infections [22]. To the best of our knowledge, our study is the first study to show that the URTI was significantly lower in the SCIT group compared with the SUC group in total subjects during three years of follow-up. Additionally, the URTI was significantly reduced compared with baseline in the SCIT group (at the two- and three-year follow-up periods for total subjects and three-year follow-up period for AR with asthma subjects). The reduction in the rate of URTI may be due to fewer symptoms of AR and asthma after receiving SCIT.

Our traditional slow desensitization SCIT showed a low incidence of a systemic reaction (0.0053 per injection), which was comparable with the previous systematic review indicating that the incidence of systemic reaction varied from 0.0074 to 0.06 per injection [23]. The incidence of systemic reaction in our traditional SCIT was lower compared with rush immunotherapy (4.7%) [24].

Our study had some strengths. Firstly, this study was designed to simulate real-world practice based on SCIT for AR subjects with or without asthma. Secondly, this study was designed for long-term follow-up for three years. However, several limitations in our study need to be mentioned. Firstly, our study could not be designed as a randomized controlled trial (RCT). Therefore, an RCT should be conducted if it is feasible to study the effectiveness of the SCIT for AR and asthma. Secondly, the direct cost in our study included only medication costs (pharmacological and SCIT costs), but did not include other indirect costs, e.g., doctor fees, transportation cost, and loss of leisure time. Thus, the cost-effectiveness including direct and indirect costs should be explored in future studies. Thirdly, this study was a retrospective study. The degrees of AR and asthma control were not mentioned. Therefore, the AR and asthma control levels should be addressed in future studies.

## 5. Conclusions

Subcutaneous immunotherapy in subjects with allergic rhinitis with or without concomitant asthma was associated with savings in terms of the cost of medication for allergic rhinitis and asthma treatment in the long term. The rates of AR and asthma exacerbations and URTI were also decreased in subjects who received SCIT.

## Figures and Tables

**Table 1:** SCIT dosage schedule.

Phase	Injection Number	Concentration	Volume (mL)	Interval
Induction	1	1:100,000	0.1	1 week
	2	1:100,000	0.2	1 week
	3	1:100,000	0.3	1 week
	4	1:100,000	0.4	1 week
	5	1:100,000	0.5	1 week
	6	1:10,000	0.1	1 week
	7	1:10,000	0.2	1 week
	8	1:10,000	0.3	1 week
	9	1:10,000	0.4	1 week
	10	1:10,000	0.5	1 week
	11	1:1000	0.1	1 week
	12	1:1000	0.2	1 week
	13	1:1000	0.3	1 week
	14	1:1000	0.4	1 week
	15	1:1000	0.5	1 week
	16	1:100	0.1	2 week
	17	1:100	0.2	2 week
Maintenance	18	1:100	0.3	4 week
	Up to 3 years	1:100	0.3	4 week

Abbreviations: SCIT, subcutaneous immunotherapy; mL, milliliter.

**Table 2:** Baseline characteristics of study participants.

Characteristics	SCIT (N = 24)	SUC (N = 16)	*p*-Value
Age (year)	34.3 ± 14.1	29.4 ± 18.1	0.333
Gender (male), *n* (%)	9 (37.5)	8 (50.0)	0.522
Body mass index (kg/m^2^)	22.4 ± 3.1	20.6 ± 3.6	0.109
Age of disease onset (year) (median, IQR)	15.0 (9.3–29.5)	17.0 (5.3–32.0)	0.795
Duration of allergic rhinitis or asthma (year) (median, IQR)	11.5 (9.0–18.3)	7.5 (5.0–8.0)	<0.001
Family history of AR or asthma, *n* (%)	18 (75.0)	12 (75.0)	1.000
Aeroallergen sensitization, *n* (%)			
Mite mixed	22 (91.7)	16 (100.0)	0.508
Cockroaches mixed	14 (58.3)	13 (81.3)	0.177
Animal dander	2 (8.3)	1 (6.2)	0.810
Grass pollen	4 (16.7)	5 (31.3)	0.441
AR with asthma, *n* (%)	15 (62.5)	10 (62.5)	1.000
Spirometry results			
%predicted of FVC	86.9 ± 9.6	89.6 ± 16.0	0.67
%predicted of FEV_1_	88.3 ± 13.7	82.4 ± 9.7	0.338
Ratio of FEV_1_ to FVC (%)	85.6 ± 7.5	80.4 ± 7.7	0.393

**Note**: Data are presented in mean ± SD or *n* (%) or otherwise stated. **Abbreviations**: IQR, interquartile range; AR, allergic rhinitis; FEV_1_, forced expiratory in the first second; FVC, forced vital capacity.

**Table 3:** Total medication cost per month (Thai baht (THB)) before and during three years of treatment between SCIT and SUC groups in AR subjects with or without asthma.

Total Subjects	SCIT (N = 24)	SUC (N = 16)	*p*-Value
Baseline	1822.5 (1504.9–2129.5)	1562.4 (1315.1–1857.3)	0.162
1st year	587.4 (348.3–1374.6) **	1562.4 (1315.1–1857.3)	<0.001
2nd year	501.2 (302.9–839.0) **	1728.3 (1190.0–2236.1)	<0.001
3rd year	372.4 (284.8–752.4) **	1500.3 (1217.9–1748.9)	<0.001
**AR with Asthma Subjects**	**SCIT (N = 15)**	**SUC (N = 10)**	***p*-Value**
Baseline	1938.5 (1725.7–2806.9)	1649.6 (1447.5–2052.7)	0.219
1st year	644.6 (432.9–1532.5) *	1649.6 (1447.5–2052.7)	0.006
2nd year	601.0 (284.7–1331.0) *	1802.9 (1303.0–2415.1)	0.001
3rd year	563.9 (350.0–937.8) *	1512.3 (1290.7–2072.8)	0.005

**Note**: Data are presented in median (IQR); *p*-value; compared between SCIT and SUC groups; **, a significant difference compared with baseline (*p* < 0.001); *, a significant difference compared with baseline (*p* < 0.01). **Abbreviations**: AR, allergic rhinitis; SCIT, subcutaneous immunotherapy; SUC, standard usual care; THB, Thai baht.

**Table 4:** AR and asthma exacerbation (time/year) required oral or systemic steroids before and after treatment between the SCIT group and SUC group in AR subjects with or without asthma.

Total Subjects	SCIT (N = 24)	SUC (N = 16)	*p*-Value
Baseline	1.0 (0.0–3.0)	0.0 (0.0–1.8)	0.084
1st year	0.0 (0.0–1.0) **	0.0 (0.0–1.8)	0.449
2nd year	0.0 (0.0–1.0) **	0.0 (0.0–1.0)	0.413
3rd year	0.0 (0.0–1.0) **	1.0 (0.0–1.0)	0.101
**AR with Asthma Subjects**	**SCIT (N = 15)**	**SUC (N = 10)**	***p*-Value**
Baseline	1.5 (0.0–3.0)	0.5 (0.0–1.3)	0.208
1st year	0.0 (0.0–1.0) *	0.5 (0.0–1.3)	0.393
2nd year	0.0 (0.0–1.0) *	0.0 (0.0–1.3)	0.58
3rd year	0.0 (0.0–0.0) *	1.0 (0.0–1.5)	0.032

**Note**: Data are presented in median (IQR); *p*-value; compared between SCIT and SUC groups; **, a significant difference compared with baseline (*p* < 0.01); *, a significant difference compared with baseline (*p* < 0.05). **Abbreviations**: AR, allergic rhinitis; SCIT, subcutaneous immunotherapy; SUC, standard usual care.

**Table 5:** Upper respiratory tract infection (URTI) (time/year) requiring antibiotics before and after treatment between the SCIT group and SUC group in AR subjects with or without asthma.

Total Subjects	SCIT (N = 24)	SUC (N = 16)	*p*-Value
Baseline	1.0 (0.0–2.0)	0.0 (0.0–2.0)	0.291
1st year	0.5 (0.0–1.8)	0.0 (0.0–2.0)	0.998
2nd year	1.0 (0.0–1.0) *	1.0 (0.0–2.8)	0.367
3rd year	0.0 (0.0–1.0) *	1.0 (0.0–3.0)	0.113
**AR with Asthma Subjects**	**SCIT (N = 15)**	**SUC (N = 10)**	***p*-Value**
Baseline	2.0 (0.0–3.3)	0.0 (0.0–2.0)	0.074
1st year	1.0 (0.0–2.0)	0.0 (0.0–2.0)	0.329
2nd year	1.0 (0.0–1.0)	0.0 (0.0–3.0)	0.615
3rd year	0.0 (0.0–1.0) *	0.0 (0.0–1.5)	0.712

**Note**: Data are presented in mean ± SD; *p*-value; compared between SCIT and SUC groups; *, a significant difference compared with baseline (*p* < 0.05). **Abbreviations**: AR, allergic rhinitis; SCIT, subcutaneous immunotherapy; SUC, standard usual care.

## Data Availability

The datasets used and/or analyzed during the current study are available from the corresponding author on reasonable request.

## References

[1] Wallace D.V., Dykewicz M.S., Bernstein D.I., Blessing-Moore J., Cox L., Khan D.A., Lang D.M., Nicklas R.A., Oppenheimer J., Portnoy J.M. (2008). The diagnosis and management of rhinitis: An updated practice parameter. J. Allergy Clin. Immunol..

[2] Pothirat C., Phetsuk N., Liwsrisakun C., Bumroongkit C., Deesomchok A., Theerakittikul T. (2016). Major Chronic Respiratory Diseases in Chiang Mai: Prevalence, Clinical Characteristics, and Their Correlations. J. Med. Assoc. Thai..

[3] Pothirat C., Chaiwong W. (2021). Aeroallergen sensitization and clinical characteristics of subjects with chronic rhinitis in Chiang Mai, Thailand: A Twenty-Year Retrospective Study. J. Asthma Allergy.

[4] Moote W., Kim H. (2011). Allergen-specific immunotherapy. Allergy Asthma Clin. Immunol..

[5] Akdis M., Akdis C.A. (2014). Mechanisms of allergen-specific immunotherapy: Multiple suppressor factors at work in immune tolerance to allergens. J. Allergy Clin. Immunol..

[6] Jutel M., Kosowska A., Smolinska S. (2016). Allergen Immunotherapy: Past, Present, and Future. Allergy Asthma Immunol. Res..

[7] Pajno G.B., Barberio G., De Luca F., Morabito L., Parmiani S. (2001). Prevention of new sensitizations in asthmatic children monosensitized to house dust mite by specific immunotherapy. A six-year follow-up study. Clin. Exp. Allergy.

[8] Purello-D’Ambrosio F., Gangemi S., Merendino R.A., Isola S., Puccinelli P., Parmiani S., Ricciardi L. (2001). Prevention of new sensitizations in monosensitized subjects submitted to specific immunotherapy or not. A retrospective study. Clin. Exp. Allergy.

[9] Droessaert V., Timmermans M., Dekimpe E., Seys S., Ceuppens J.J., Fokkens W.J., Hellings P.W. (2016). Real-life study showing better control of allergic rhinitis by immunotherapy than regular pharmacotherapy. Rhinology.

[10] Lu Y., Xu L., Xia M., Li Y., Cao L. (2015). The efficacy and safety of subcutaneous immunotherapy in mite-sensitized subjects with asthma: A meta-analysis. Respir. Care.

[11] Targowski T., Jahnz-Rózyk K., Przekora P., Kucharczyk A., Owczarek W. (2011). Clinical efficacy and costs of the three-year specific allergen immunotherapy—Retrospective study. Pol. Merkur. Lekarski..

[12] Tu Y., Zhang H., Zhao L., Jin P., Zi X., Li T., Shi L., Zhi L. (2019). The changes in different symptom scores during subcutaneous immunotherapy in Chinese house dust mite allergic patients: A two-year, observational study. J. Laryngol. Otol..

[13] Dominguez-Ortega J., Delgado J., Blanco C., Prieto L., Arroabarren E., Cimarra M., Henriquez-Santana A., Iglesias-Souto J., Vega-Chicote J.M., Tabar A.I. (2017). Specific allergen immunotherapy for the treatment of allergic asthma: A review of current evidence. J. Investig. Allergol. Clin. Immunol..

[14] Abramson M.J., Puy R.M., Weiner J.M. (2000). Allergen immunotherapy for asthma. Cochrane Database Syst. Rev..

[15] Asaria M., Dhami S., van Ree R., van Wijk G.R., Muraro A., Roberts G., Sheikh A. (2018). Health economic analysis of allergen immunotherapy for the management of allergic rhinitis, asthma, food allergy and venom allergy: A systematic overview. Allergy.

[16] Rottem M., Egbarya A. (2014). Subcutaneous immunotherapy in Northern Israel: Efficacy and safety. Isr. Med. Assoc. J..

[17] Petersen K.D., Gyrd-Hansen D., Dahl R. (2005). Health-economic analyses of subcutaneous specific immunotherapy for grass pollen and mite allergy. Allergol. Immunopathol..

[18] Thanborisutkul K., Khodtecha N., Kulalert P., Sritipsukho P. (2020). Efficacy, patients’ perception, and cost of medication in allergic rhinitis with subcutaneous immunotherapy. Asian Pac. J. Allergy Immunol..

[19] Rantala A., Jaakkola J.J., Jaakkola M.S. (2013). Respiratory infections in adults with atopic disease and IgE antibodies to common aeroallergens. PLoS ONE.

[20] Reinhold T., Ostermann J., Thum-Oltmer S., Brüggenjürgen B. (2013). Influence of subcutaneous specific immunotherapy on drug costs in children suffering from allergic asthma. Clin. Transl. Allergy.

[21] Pifferi M., Baldini G., Marrazzini G., Baldini M., Ragazzo V., Pietrobelli A., Boner A.L. (2002). Benefits of immunotherapy with a standardized Dermatophagoides pteronyssinus extract in asthmatic children: A three-year prospective study. Allergy.

[22] Gilmour M.I. (2012). Influence of air pollutants on allergic sensitization: The paradox of increased allergies and decreased resistance to infection. Toxicol. Pathol..

[23] Dhami S., Kakourou A., Asamoah F., Agache I., Lau S., Jutel M., Muraro A., Roberts G., Akdis C.A., Bonini M. (2017). Allergen immunotherapy for allergic asthma: A systematic review and meta-analysis. Allergy.

[24] Fajt M.L., Rosenberg S.L., Yecies E., Traister R.S., Petrov A.A. (2017). A 10-year experience of a novel and safe modified environmental rush immunotherapy protocol. Allergy Asthma Proc..

